# Clinical Outcomes of Combined Phacoemulsification, Extended Depth-of-Focus Intraocular Lens Implantation, and Epiretinal Membrane Peeling Surgery

**DOI:** 10.3390/jcm14072423

**Published:** 2025-04-02

**Authors:** Ho-Seok Chung, Dabin Lee, Jin-Hyoung Park

**Affiliations:** 1 Department of Ophthalmology, Asan Medical Center, Ulsan University College of Medicine, Seoul 05505, Republic of Korea; chunghoseok@gmail.com; 2 Department of Ophthalmology, Dankook University Hospital, Dankook University College of Medicine, Cheonan 31116, Republic of Korea; dabong1118@gmail.com; 3 MS Eye Clinic, Seoul 06512, Republic of Korea

**Keywords:** cataract, epiretinal membrane, extended depth-of-focus intraocular lens, presbyopia

## Abstract

**Background/Objectives**: To evaluate the clinical efficacy and safety of combined phacoemulsification, extended depth-of-focus (EDOF) intraocular lens (IOL) implantation, and epiretinal membrane (ERM) peeling during vitrectomy surgery for treating patients with ERM, cataracts, and presbyopia. **Methods**: Patients with preexisting low-grade ERM who underwent cataract surgery with the implantation of an EDOF IOL were included. Corrected distance visual acuity (CDVA), uncorrected distance visual acuity (UDVA), uncorrected intermediate visual acuity (UIVA), uncorrected near visual acuity (UNVA), autorefraction and keratometry, manifest refraction, and central foveal thickness (CFT) were measured before surgery and at postoperative months 3 and 6. A monocular defocus curve was measured 6 months postoperatively. Furthermore, patients were instructed to report symptoms of photic phenomena at each visit. **Results**: In total, 16 eyes of 16 patients (median age, 59.5 years) were included in this study. Compared with those at baseline, the CDVA, UDVA, UIVA, UNVA, and CFT significantly improved at 3 and 6 months postoperatively. The defocus curve revealed that a visual acuity of 0.12 logarithm of the minimal angle of resolution or better was maintained from +0.5 to –1.5 diopters. No patients reported visual disturbances suggestive of photic phenomena, such as glare or halo. **Conclusions**: EDOF IOL implantation had excellent outcomes, including improved distance and intermediate visual acuity, functional near visual acuity, and absence of visual symptoms in patients who received phacovitrectomy to treat low-grade ERM.

## 1. Introduction

The prevalence of presbyopia is increasing along with the increase in life expectancy; this has led to a greater demand for improved near vision [1,2]. The goal of cataract surgery is to provide vision rehabilitation as well as to provide improved distance vision, if possible, including acceptable intermediate and near vision [3,4]. Many studies have reported improved visual acuity, glasses independence, and patient satisfaction with the use of multifocal intraocular lenses (IOLs) rather than near-targeted monofocal IOLs [5]. Despite the increased popularity of multifocal IOLs for cataract surgery, several contraindications, particularly macular pathologies, have been a barrier to their use. However, positive clinical findings from multifocal IOL implantation in patients with retinal disease have recently been reported [6,7,8].

Epiretinal membrane (ERM) is a cellular proliferative disease of the inner retinal surface. According to an epidemiological study of ERM in Korean populations, the overall prevalence of ERM in individuals aged 40 years or older was 2.9%, with further increases in elderly individuals [9]. The clinical efficacy and safety of phacovitrectomy, the combination of phacoemulsification with monofocal IOL implantation and pars plana vitrectomy (PPV) with the ERM peeling procedure, have been reported in many studies [10,11,12]. Due to the benefits of a shorter healing time, lower surgical cost, and simultaneous removal of expected cataract progression following vitrectomy, phacovitrectomy has become a common procedure in the surgical management of ERM and cataracts. In addition, a prior study reported good distance and near vision resulting from the implantation of a diffractive multifocal IOL with phacovitrectomy in patients with low-grade ERM [13].

Extended depth-of-focus (EDOF) IOLs are designed to provide an extended range of intermediate vision with functional near vision by utilizing a unique light diffraction pattern to extend the eye’s focal range [14]. The Tecnis Symfony (Abbott Medical Optics, Abbott Park, IL, USA), previously classified as an EDOF IOL, has been reclassified under a functional classification system as a Partial Range of Field-Extended (PRoF-Ex) IOL [15]. PRoF-Ex IOLs exhibit a smooth, gradual decrease in visual acuity across the defocus curve without sharp focal peaks, maintaining intermediate vision over a broad range. Tecnis Symfony IOL incorporates an aspheric or toric-aspheric anterior surface optic and a diffractive design, known as Echolette technology, on the posterior surface [16,17]. This IOL has a C-loop haptic with intermediate added power (+1.75 diopter [D]) in the IOL plane. One study reported no statistically significant differences in distance or intermediate visual acuity between EDOF IOLs and diffractive trifocal IOLs [18]. Moreover, studies have reported that the contrast sensitivity of EDOF IOLs is higher than that of trifocal IOLs, especially under scotopic conditions [19,20].

To our knowledge, no studies have reported on the clinical outcomes of simultaneous phacovitrectomy and EDOF IOL implantation during surgery for ERM. Therefore, the present study aimed to evaluate the clinical efficacy and safety of combined phacoemulsification, EDOF IOL implantation, and PPV with ERM peeling surgery in patients with ERM, cataracts, and presbyopia.

## 2. Materials and Methods

### 2.1. Patients

This retrospective case series included patients who had undergone combined phacoemulsification and vitrectomy. The review included the eyes of patients treated at the MS Eye Clinic from December 2018 to July 2022. The study was approved by the Institutional Review Board of Dankook University Hospital (IRB no. 2022-07-022) on 24 August 2022. Informed consent was waived because of the retrospective nature of this study. The study was conducted in accordance with the tenets of the Declaration of Helsinki and all federal laws. The study included eyes with preexisting ERM diagnosed by spectral-domain optical coherence tomography (SD-OCT, Canon OCT-HS100, Tokyo, Japan) that underwent simultaneous phacoemulsification cataract extraction, EDOF IOL implantation, and PPV with ERM peeling. The exclusion criteria included eyes with a history of ocular surgery, including refractive surgery; corneal astigmatism greater than 1.5 D; a history of macular pathologies other than ERM; severe dry eye disease or abnormal high-order aberrations, which could have affected postoperative visual outcomes; and eyes for which 3- and 6-month postoperative follow-up data were unavailable.

### 2.2. ERM Classification

ERM stages were defined on SD-OCT scans as follows, as previously described by Govetto et al. [21]: (i) stage 1: mild, thin ERMs with a preserved foveal depression and distinct retinal layers; (ii) stage 2: loss of foveal depression with the widening of the outer nuclear layer; retinal layers remain distinct; (iii) stage 3: presence of continuous ectopic inner foveal layers crossing the entire foveal area; retinal layers are still distinguishable; and (iv) stage 4: thick ERMs with disrupted macular architecture, indistinguishable retinal layers, and continuous ectopic inner foveal layers. This study included patients with stage 1 and 2 ERM.

Our criteria for performing ERM peeling surgery included the following: First, if a low-grade ERM was found in preoperative examinations of a patient planning to undergo cataract surgery with EDOF IOL implantation, the possibility of dysphotopsia after cataract surgery was discussed with the patient, and a combined phacovitrectomy surgery with ERM peeling was planned. Next, among patients with symptomatic ERM due to metamorphopsia or reduced contrast sensitivity who wanted to improve near vision because of presbyopia, a combined phacovitrectomy surgery with EDOF IOL implantation was performed in a single surgical session.

### 2.3. Surgical Procedure

Peribulbar anesthesia was used for all procedures. The uncomplicated phacoemulsification technique was performed through a 2.2 mm incision, and an EDOF IOL (TECNIS^®^ Symfony ZXR00, Johnson and Johnson Surgical Vision, Inc., Irvine, CA, USA) was implanted. The IOL power was calculated using the Barrett Universal II formula and an IOL Master 700 (Carl Zeiss Meditec, Dublin, CA, USA), and emmetropia was targeted. After placing and centering the IOL in the capsular bag, all viscoelastic materials were removed from the anterior chamber. The incision was closed with a 10-0 nylon suture. After cataract surgery, 23-gauge PPV was performed using the Constellation Vitrectomy System^®^ (Alcon Laboratories, Inc., Fort Worth, TX, USA) machine through a noncontact wide-angle viewing system (Occulus BIOM^®^ surgical systems, OCULUS, Wetzlar, Germany). Standard techniques were used for membrane peeling of the ERM, with the removal of the internal limiting membrane (ILM) assisted by indocyanine green dye mixed in a 5% dextrose solution. No gas was used during the surgery. All surgeries were performed by a single vitreoretinal surgeon (JHP).

### 2.4. Clinical Examinations

The data reviewed from medical charts included those obtained via clinical examinations performed preoperatively and at 3 and 6 months postoperatively. The preoperative examination included corrected distance visual acuity (CDVA), uncorrected distance visual acuity (UDVA), uncorrected intermediate visual acuity (UIVA), uncorrected near visual acuity (UNVA), non-contact tonometer (TX-20P; Canon, Tokyo, Japan), autorefraction and keratometry (Topcon KR700, Japan), manifest refraction, and central foveal thickness (CFT). The postoperative examinations (3 and 6 months) included CDVA, UDVA, UIVA, UNVA, non-contact tonometer, autorefraction, keratometry, manifest refraction, and CFT. Patients were also asked whether they had experienced metamorphopsia, visual distortion, or dysphotopsia at each postoperative visit (for example, visual symptoms such as halo, flicker, and glare).

Distance (5 m), intermediate (66 cm), and near (33 cm) visual acuity were measured. Distance visual acuity was measured using Hahn’s standard test chart, and intermediate visual acuity and near visual acuity were measured using a Jaeger standard test chart. For the statistical analyses, each visual acuity was converted into the logarithm of the minimal angle of resolution (logMAR).

CFT was defined as the distance measured at the thickest point of the foveal region from the retinal pigment epithelium to the ILM. SD-OCT software (Rx Capture Version 4.4.0.11), including the caliper tool, was used to measure the CFT.

A monocular defocus curve was obtained 6 months postoperatively. For distance correction, visual acuity was estimated at 5 m, and defocusing and lens addition were performed from −3.5 D to +2.0 D in 0.5-D steps.

### 2.5. Statistical Analyses

The Shapiro–Wilk test was used to assess the distribution of the numerical data. The data are expressed as the median (interquartile range [IQR]). The Friedman test with post hoc Bonferroni correction was used to investigate changes in visual acuity and CFT before and then at three and six months after surgery. The Wilcoxon signed-rank test evaluated post hoc differences in clinical findings before and after surgery. Statistical analyses were conducted using SPSS software (25.0, SPSS Inc., Chicago, IL, USA). *p* values < 0.05 were considered to indicate significance.

## 3. Results

Sixteen eyes of 16 patients were included. The baseline characteristics and preoperative clinical findings of all patients are summarized in Table 1. Four male and ten female patients (median age 59.5 years) were included. During the follow-up period, no significant postoperative complications, including cystoid macular edema, endophthalmitis, or hypotony, were reported. No patients reported visual disturbances such as glare, halo, or starburst in daily activities during the follow-up period.

**Figure 1:**
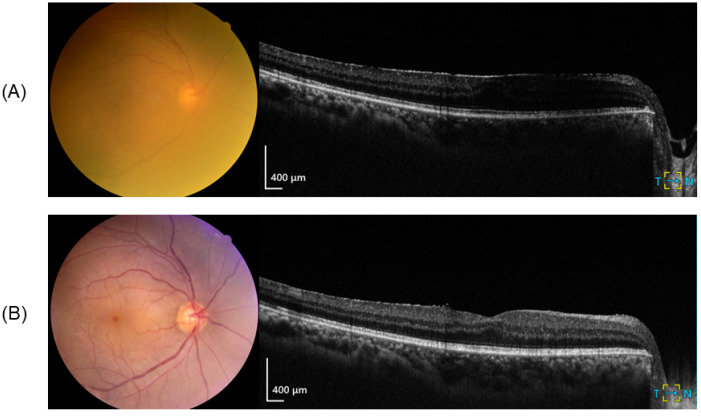
Representative case. (**A**) Preoperative fundus photography and optical coherence tomography and (**B**) postoperative fundus photography and optical coherence tomography. The preoperative central foveal thickness was 381 μm, and the postoperative central foveal thickness was 344 μm.

Table 2 presents the detailed clinical findings before and after surgery, including refractive error, visual acuity at various distances, and central foveal thickness. All patients exhibited a UDVA of 0.10 logMAR or better, a UIVA of 0.22 logMAR or better, and a UNVA of 0.4 logMAR or better at 6 months postoperatively. Figure 1 shows a representative case (patient 5) of pre- and postoperative fundus photography and OCT. Before surgery, blurred fundus photos due to cataracts and central foveal thickening (CFT 381 μm) due to stage 2 ERM were observed. At 6 months postoperatively, the fundus was more clearly observable, the foveal depression had partially recovered, and the central foveal thickening had also improved (CFT 344 μm).

Table 3 presents the visual function and structural outcomes preoperatively and at 3 and 6 months postoperatively. The UDVA at 3 months postoperatively was 0.05 (IQR 0.08) logMAR, which significantly decreased from 0.40 (IQR 0.22) logMAR preoperatively (adjusted *p* < 0.001). The UDVA at 6 months postoperatively was 0.0 (IQR 0.03) logMAR, which was significantly improved compared to the preoperative UDVA (adjusted *p* < 0.001). The UIVA measured 3 months postoperatively was 0.19 (IQR 0.09) logMAR, and the UIVA measured 6 months postoperatively was 0.10 (IQR 0.09) logMAR. The UIVAs at 3 and 6 months postoperatively were significantly improved relative to the preoperative baseline (adjusted *p* = 0.003 at 3 and 6 months postoperatively). The UNVA at 3 months postoperatively was 0.30 (IQR 0.19) logMAR, which significantly decreased from 0.60 (IQR 0.21) logMAR at the preoperative baseline (adjusted *p* = 0.003). The UNVA at 6 months postoperatively was 0.30 (IQR 0.11) logMAR, which was significantly improved relative to the preoperative UNVA (adjusted *p* < 0.001).

The spherical equivalent was −0.18 (IQR 1.94) D preoperatively, 0.0 (IQR 0.0) D at 3 months postoperatively, and 0.0 (IQR 0.0) D at 6 months postoperatively. The pre- and postoperative spherical equivalents were not significantly different. The final postoperative spherical equivalent was within ±0.38 D in all patients. The preoperative CFT was 362.5 (IQR 58.5) µm, and the CFT 3 months postoperatively was 351.0 (IQR 61.8) µm, indicating a significant decrease (adjusted *p* = 0.009). At 6 months postoperatively, the CFT was 329.5 (IQR 55.0) µm, indicating a significant decrease relative to the preoperative CFT (adjusted *p* < 0.001), and all patients experienced decreased CFT at 6 months postoperatively compared with the preoperative CFT.

Table 4 presents the proportions of patients with CDVA, UDVA, UIVA, and UNVA at 3 and 6 months postoperatively. Up to 43.8% of patients had a UDVA of 0.0 logMAR or better at 3 months postoperatively, and 75.0% had a UDVA of 0.0 logMAR or better at 6 months postoperatively. At 3 months postoperatively, 37.5% of patients had a UNVA of 0.20 or better, and 43.8% showed this improvement at 6 months postoperatively.

**Figure 2:**
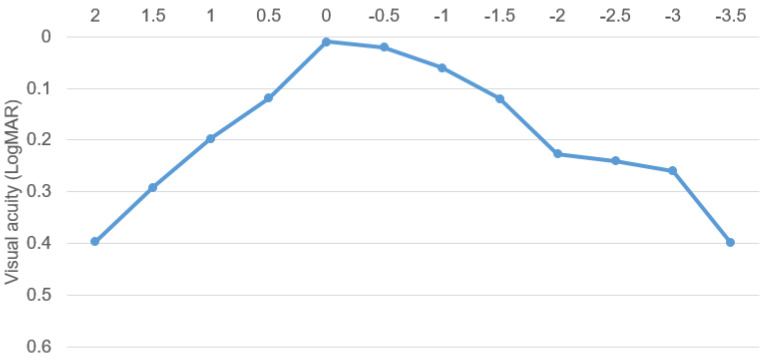
Defocus curve from –3.5 diopters to 2.0 diopters. A visual acuity of 0.12 logMAR or better was obtained over a wide range from +0.50 diopter to −1.50 diopter, and a visual acuity of 0.4 logMAR or better was obtained in all diopters performed.

Figure 2 shows the monocular defocus curve at 6 months. The monocular defocus curve indicated continuous visual performance and no visual acuity gap for intermediate-range vision. The best visual acuity was at 0.0 D defocus (0.0 logMAR), a visual acuity of 0.12 logMAR or better was obtained over a wide range from +0.50 D to −1.50 D, and a visual acuity of 0.4 logMAR or better was obtained in all diopters performed.

## 4. Discussion

The present study demonstrated the efficacy and safety of EDOF IOL implantation conducted concurrently with vitrectomy in 16 patients with low-grade ERM and cataracts. The implantation of the EDOF IOL during phacovitrectomy resulted in excellent distance and intermediate visual acuity and acceptable near visual acuity, with no significant photic phenomena. The defocus curve revealed a broad intermediate-near range and defocus tolerance, similar to findings after EDOF IOL implantation in nonvitrectomized eyes. Our study confirms that the Tecnis Symfony IOL aligns with the PRoF-Ex classification, supporting the updated functional categorization of IOLs [15]. Compared to traditional EDOF lenses, PRoF-Ex IOLs provide a more gradual decline in visual acuity across the defocus curve, enhancing intermediate vision without the pronounced focal peaks seen in multifocal IOLs [15]. Although this was a case series with a small sample size, it reported positive clinical outcomes, suggesting the well-tolerated use of EDOF IOLs in eyes with macular disease, which has been relatively contraindicated.

The use of multifocal IOLs in eyes with retinal disease should be considered with caution because it can interfere with patients’ daily lives [22]. Multifocal IOLs can potentially exacerbate contrast sensitivity impairments in retinal disease due to the nature of light splitting, and patients may experience visual symptoms such as halo, flicker, and glare [23]. Patel et al. reported improved distance visual acuity and good near vision (J2 or better) in six eyes that underwent bifocal diffractive IOL implantation with phacovitrectomy [8]. However, that study included only patients with symptomatic vitreous floaters, while the present study included patients with macular structural abnormalities that caused metamorphopsia, suggesting a wider indication for the use of multifocal IOLs [8]. A recent study evaluated 20 pseudophakic eyes implanted with diffractive trifocal IOLs that underwent vitrectomy for ERM, showing good visual recovery after 12 months [24]. However, unlike trifocal IOLs that provide discrete focal points, EDOF IOLs offer a smoother visual transition and may reduce the risk of photic disturbances. Additionally, EDOF IOLs may better preserve contrast sensitivity than trifocal IOLs, making them preferable for ERM patients. In contrast, trifocal IOLs may cause further contrast reduction in ERM patients [19,20]. These findings suggest that while both trifocal and EDOF IOLs can be used in ERM patients, EDOF IOLs may be preferable due to their extended functional range and lower likelihood of visual disturbances.

In this study, excellent visual outcomes at far and intermediate distances and functional near vision indicated expectations for spectacle independence with the use of EDOF IOLs. All visual acuity outcomes exhibited statistically significant improvements at 3 and 6 months postoperatively compared to the preoperative baseline values. The UIVA was 0.10 (IQR 0.09) at 6 months, and all patients exhibited stable improvement of 0.22 logMAR or better. EDOF IOLs are effective at far and intermediate distances but less effective at near distances than other multifocal IOL designs [25,26]. However, some studies have reported satisfactory quality of near vision with EDOF IOLs, and this issue remains controversial [27,28]. In the present study, 43.8% of patients had a UNVA of 0.2 logMAR or better at 6 months postoperatively, suggesting that functional near visual acuity was achieved in almost half of the patients. The performance of EDOF IOLs implanted simultaneously with phacovitrectomy was not significantly different from that of nonvitrectomized eyes, as indicated by the defocus curve. A large defocus tolerance was confirmed with 0.12 logMAR or better visual acuity sustained from +0.5 D to −1.5 D, and a visual acuity of 0.4 logMAR or better sustained through −2.5 D indicated a wide intermediate-near range, aligning with the PRoF-Ex category [15]. In patients with mild ERM without foveal involvement, excellent results have also been reported with phacoemulsification and multifocal IOL implantation without vitrectomy [29]. However, previous studies have suggested that this technique is only applicable in carefully selected cases, and our results suggest good visual outcomes even if ILM peeling is performed together with IOL implantation.

Interventions in the present study had an excellent safety profile, as no patients reported visual disturbances such as glare, halo, or starburst during daily activities. Generally, EDOF IOLs and diffractive multifocal IOLs, whether bifocal or trifocal, cause mild to moderate visual disturbances, such as glare and halos. However, EDOF IOLs are known to have a lower incidence of halos than both bifocal and trifocal IOLs, which is consistent with the outcomes reported in our study [30,31]. However, this was only a result of inquiring about whether they felt discomfort due to photic phenomena and not about the degree of discomfort. Therefore, patients experiencing mild visual discomfort might not have been included.

The spherical equivalent at 6 postoperative months was 0.0 (IQR 0.0) D, and all patients exhibited a refractive error within ±0.38 D. In a previous study, posterior capsular opacity and ametropia accounted for >80% of patient dissatisfaction after multifocal IOL implantation. Moreover, it has been reported that combined phacovitrectomy can result in greater postoperative refractive error compared to that associated with phacoemulsification alone following vitrectomy [32,33]. In these cases, the calculation of IOL power with axial length measured by IOL Master and ultrasound biometry revealed a significant postoperative myopic shift following combined phacovitrectomy [34]. Conversely, another study reported no tendency for myopic shift when IOL power was calculated using the IOL Master [35]. This possibility of residual refractive error may influence surgeons’ decisions to decrease the use of multifocal IOLs or to conduct combined phacovitrectomy less frequently. However, many studies have reported that the Tecnis Symfony IOLs exhibit excellent tolerance to unexpected refractive errors compared to other multifocal IOLs and do not significantly change monocular or binocular corrected visual acuity, which has additional value in multiple clinical situations [36,37,38].

Another obstacle to the use of multifocal IOLs in retinal diseases is the intraoperative difficulty of retinal visualization during PPV. Yoshino et al. reported that diffractive multifocal IOLs affected the surgical field of view, causing intraoperative difficulty [39]. However, other studies have reported no intraoperative difficulty with the surgical field of view during combined phacovitrectomy [30,40,41]. In the present study, the surgeon did not experience difficulty in focusing when performing core vitrectomy or ILM peeling. However, because the experience and proficiency of surgeons vary and precise procedures such as ILM peeling are often required for the surgical management of ERM patients, additional research with a larger number of patients is required to optimize the securing of the surgical field of view when multifocal IOLs are used. The EDOF IOL used in the present study had fewer diffractive rings and was, therefore, less likely to adversely affect intraoperative retinal visualization than other IOL models, such as the ZM900^®^ (Abbott Medical Optics, Johnson and Johnson Vision), which caused visualization difficulties during PPV surgery in a previous study [39].

The present study was limited by its retrospective case series design. We did not measure contrast sensitivity or use a standardized questionnaire to evaluate patients’ subjective visual quality, such as photic phenomena or satisfaction. We relied on descriptions of visual discomfort written in medical records. Considering that the occurrence of halos is associated with reduced contrast sensitivity, questions about visual symptoms may be somewhat predictive of a severe reduction in contrast sensitivity [42]. However, our results pertaining to contrast sensitivity and adverse effects may have limited interpretability. Another limitation is that the angle alpha, which has been reported to be useful in predicting subjective satisfaction and the occurrence of photic phenomena in a previous study, was not measured [43]. The small sample size and absence of a control group, such as age-matched patients, make the statistical power insufficient to analyze efficacy and safety. Future prospective studies with more patients are needed to better evaluate the clinical utility of the EDOF IOL, including a direct comparison with monofocal or trifocal diffractive multifocal IOLs in combined phacovitrectomy.

## 5. Conclusions

In conclusion, when combined with phacovitrectomy to treat patients with low-grade ERM, EDOF IOL implantation provides excellent outcomes in terms of distance and intermediate visual acuity, as well as functional near visual acuity. In ERM patients who experience discomfort from presbyopia, EDOF IOL implantation in combined phacovitrectomy is potentially beneficial for removing the ERM and obtaining spectacle independence.

## Figures and Tables

**Table 1:** Baseline characteristics of the study patients.

N = 16	Median (IQR)
Age (years)	59.5 (9.5)
Sex (M:F)	4:12
Spherical equivalent (diopters)	−0.18 (1.94)
Intraocular pressure (mmHg)	17.5 (3.75)
CDVA (logMAR)	0.22 (0.07)
UDVA (logMAR)	0.40 (0.22)
UIVA (logMAR)	0.40 (0.22)
UNVA (logMAR)	0.60 (0.21)
CFT (μm)	362.5 (58.5)

IQR = interquartile range; CDVA = corrected distance visual acuity; UDVA = uncorrected distance visual acuity; UIVA = uncorrected intermediate visual acuity; UNVA = uncorrected near visual acuity; CFT = central foveal thickness; logMAR = logarithm of the minimal angle of resolution.

**Table 2:** Summary of pre- and postoperative findings of the study patients.

No	Age/Sex	Preop CDVA	Preop IOP (mmHg)	Preop CFT (μm)	Postop SE (D)	Postop UDVA	Postop UIVA	Postop UNVA	Postop IOP (mmHg)	Postop CFT (μm)
1	59/F	0.22	18	364	–0.25	0.05	0	0.30	16	321
2	58/F	0.15	17	326	0	0	0.22	0.40	15	309
3	58/M	0.15	15	332	+0.38	0	0.19	0.40	15	312
4	52/F	0.22	18	405	0	0	0.10	0.19	15	393
5	60/M	0.22	20	381	+0.25	0.05	0.22	0.30	19	344
6	59/F	0.22	16	426	0	0	0.10	0.19	13	388
7	67/F	0.22	18	389	–0.38	0	0.10	0.19	17	344
8	66/M	0.10	13	361	0	0	0.10	0.30	11	334
9	55/F	0.15	18	332	0	0	0.10	0.30	15	315
10	68/F	0.10	14	338	0	0	0.00	0.19	16	313
11	63/M	0.15	14	345	0	0	0.10	0.10	14	307
12	53/F	0.15	17	350	–0.25	0	0.10	0.19	15	325
13	54/F	0.40	15	364	0	0	0.10	0.30	12	340
14	68/F	0.30	19	393	0	0.10	0.22	0.30	18	375
15	61/F	0.22	19	323	0	0	0.10	0.19	18	312
16	61/F	0.30	20	419	0	0.10	0.19	0.40	17	394

All visual acuities are expressed as logMAR and postoperative findings were measured at 6 months after surgery. CDVA = corrected distance visual acuity; CFT = central foveal thickness; SE = spherical equivalent; D = diopters; UDVA = uncorrected distance visual acuity; UIVA = uncorrected intermediate visual acuity; UNVA = uncorrected near visual acuity; logMAR = logarithm of the minimal angle of resolution; IOP = intraocular pressure.

**Table 3:** Visual and structural outcomes preoperatively and at 3 and 6 months postoperatively.

	Baseline	3 Months	6 Months
Spherical equivalent (diopters)	−0.18 (1.94) (range −9.5–1.0)	0.0 (0.0) (range −0.5–0.5)	0.0 (0.0) (range −0.38–0.38)
CDVA (logMAR)	0.22 (0.07)	0.05 (0.10)	0.0 (0.03)
UDVA (logMAR)	0.40 (0.22)	0.05 (0.08)	0.0 (0.03)
UIVA (logMAR)	0.40 (0.22)	0.19 (0.09)	0.10 (0.09)
UNVA (logMAR)	0.60 (0.21)	0.30 (0.19)	0.30 (0.11)
CFT (μm)	362.5 (58.5)	351.0 (61.8)	329.5 (55.0)

All measurements are expressed as medians (interquartile ranges). CDVA = corrected distance visual acuity; UDVA = uncorrected distance visual acuity; UIVA = uncorrected intermediate visual acuity; UNVA = uncorrected near visual acuity; CFT = central foveal thickness; logMAR = logarithm of the minimal angle of resolution.

**Table 4:** Percentages of eyes within the specified CDVA, UDVA, UIVA, and UNVA values at 3 and 6 months postoperatively.

	Parameter (logMAR)	0.0 or Better	0.10 or Better	0.20 or Better	0.30 or Better	0.40 or Better	0.50 or Better
3 months	CDVA	43.8%	100%	100%	100%	100%	100%
	UDVA	37.5%	100%	100%	100%	100%	100%
	UIVA	0%	43.8%	81.3%	93.8%	100%	100%
	UNVA	0%	0%	37.5%	37.5%	93.8%	100%
6 months	CDVA	75.0%	100%	100%	100%	100%	100%
	UDVA	75.0%	100%	100%	100%	100%	100%
	UIVA	12.5%	68.8%	81.3%	100%	100%	100%
	UNVA	0%	6.3%	43.8%	43.8%	100%	100%

CDVA = corrected distance visual acuity; UDVA = uncorrected distance visual acuity; UIVA = uncorrected intermediate visual acuity; UNVA = uncorrected near visual acuity; logMAR = logarithm of the minimal angle of resolution.

## Data Availability

The dataset used during the current study is available from the corresponding author upon reasonable request.
